# 25-Hydroxyvitamin D and Parathyroid Hormone Levels Are Independently Associated with the Hemoglobin A_1c_ Level of Korean Type 2 Diabetic Patients: The Dong-Gu Study

**DOI:** 10.1371/journal.pone.0158764

**Published:** 2016-06-30

**Authors:** Seong-Woo Choi, Sun-Seog Kweon, Young-Hoon Lee, So-Yeon Ryu, Jin-Su Choi, Jung-Ae Rhee, Hae-Sung Nam, Seul-Ki Jeong, Kyeong-Soo Park, Hee Nam Kim, Min-Ho Shin

**Affiliations:** 1 Department of Preventive Medicine, Chosun University Medical School, 309, Pilmun-daero, Dong-gu, Gwangju 501–759, Republic of Korea; 2 Department of Preventive Medicine, Chonnam National University Medical School, 160, Baekseo-ro, Dong-gu, Gwangju 501–746, Republic of Korea; 3 Jeonnam Regional Cancer Center, Chonnam National University Hwasun Hospital, 322, Seoyang-ro, Hwasun, Jeollanamdo 519–809, Republic of Korea; 4 Department of Preventive Medicine & Institute of Wonkwang Medical Science, Wonkwang University School of Medicine, 344–2 Shinyong-dong, Iksan, Jeollabukdo 570–711, Republic of Korea; 5 Department of Preventive Medicine, Chungnam National University Medical School, Munhwa 1(il)-dong, Jung-gu, Daejeon 301–747, Republic of Korea; 6 Department of Neurology & Research Institute of Clinical Medicine, Biomedical Institute of Chonbuk National University Hospital, Chonbuk National University, San 2–20, Geumam-dong, Deokjin-gu, Jeonju, Jeollabukdo 561–180, Republic of Korea; 7 Department of Preventive Medicine, Seonam University College of Medicine, 439, Chunhyang-ro, Namwon, Jeollabukdo 590–711, Republic of Korea; 8 Center for Creative Biomedical Scientists, Chonnam National University, 77 Yongbong-ro, Buk-gu, Gwangju 500–757, Republic of Korea; University of Alabama at Birmingham, UNITED STATES

## Abstract

In type 2 diabetic patients, the relationships between 25-hydroxyvitamin D and parathyroid hormone levels, and glycemic control, remain unclear. We evaluated associations between 25-hydroxyvitamin D, parathyroid hormone, and hemoglobin A_1c_ levels after adjusting for other covariates, including log transformed 25-hydroxyvitamin D levels and log transformed parathyroid hormone levels, in Korean patients with type 2 diabetes. In total, 1,175 patients with type 2 diabetes were selected from 8,857 individuals who completed the baseline survey of the Dong-gu study, conducted in Korea from 2007 to 2010. After adjusting for other covariates, we found that the mean hemoglobin A_1c_ level was inversely associated with the 25-hydroxyvitamin D level (Q1: 7.47% [7.30–7.63], Q2: 7.25% [7.09–7.40], Q3: 7.17% [7.02–7.32], Q4: 7.19% [7.02–7.35]; *p* for trend = 0.021, *p* for between groups = 0.050) and the parathyroid hormone level (Q1: 7.35% [7.19–7.51], Q2: 7.34% [7.19–7.50], Q3: 7.28% [7.13–7.43], Q4: 7.09% [6.94–7.24]; *p* for trend = 0.022, *p* for between groups = 0.048). However, the mean fasting glucose level was not associated with either the 25-hydroxyvitamin D or parathyroid hormone level. In conclusion, inverse associations were evident between hemoglobin A_1c_, 25-hydroxyvitamin D and parathyroid hormone levels in Korean patients with type 2 diabetes. The associations remained significant after adjusting for other covariates, including the log transformed 25-hydroxyvitamin D levels and log transformed parathyroid hormone levels.

## Introduction

Diabetes and associated complications are becoming major public health problems. Diabetes affected 422 million subjects in 2014 and total deaths caused by high blood glucose levels in 2012 numbered 3.7 million [1]. Thus, improvement of glycemic control is an increasingly important clinical issue.

Some 80–90% of vitamin D is produced from skin 7-dehydrocholesterol upon adequate exposure to ultraviolet light; only 10–20% is derived from dietary sources such as oily fish, milk, butter, eggs, and various supplements [2]. Vitamin D is first hydroxylated in the liver to 25-hydroxyvitamin D (25[OH]D), which is the major circulating form; it is then further hydroxylated in the kidney, to 1,25-dihydroxyvitamin D (1,25[OH]_2_D), which is the biologically active form [3]. 1,25(OH)_2_D is known to regulate glucose metabolism by interacting with the 1-alpha-hydroxylase enzyme and the vitamin D receptor (VDR) in pancreatic β-cells [4,5]. Vitamin D plays an important role in bone and mineral metabolism, and a deficiency thereof is associated with the development of metabolic bone diseases including rickets and osteomalacia [6]. Recently, several health problems have been associated with absence of the non-skeletal actions of vitamin D [7,8].

However, no consensus on any association between vitamin D level and glycemic control has been attained in either observational studies or clinical trials featuring vitamin D supplementation [9]. Several authors have reported inverse associations between the 25(OH)D level and the effectiveness of glycemic control [10,11]; others found no such associations [12]. Moreover, the effect of parathyroid hormone (PTH), a known mediator of vitamin D action, was not considered.

Most previous studies sought to explain the relationship between vitamin D and PTH in the context of hypovitaminosis D and secondary hyperparathyroidism; thus, data on the independent effects of vitamin D and PTH on the HbA_1c_ levels of general populations that do not exhibit hypovitaminosis D are lacking. Koreans are of particular interest because the overall prevalence of vitamin D deficiency in Korea is 1.3-to-1.5-fold greater than in the USA, and the burden of diabetes is increasing in Korea [13]. Therefore, we sought independent associations between 25(OH)D, PTH, and HbA_1c_ levels in Korean patients with type 2 diabetes.

## Materials and Methods

### Subjects

The Dong-gu study is an ongoing prospective population-based study investigating the prevalence and incidence of, and risk factors for, chronic disease in an elderly urban population. The Dong-gu study complies with all tenets of the Declaration of Helsinki and was approved by the Institutional Review Board of Chonnam National University Hospital. All participants gave written informed consent. Detailed information on the study participants and the measurements performed in relation to this population have been previously published [14]. National resident registration records were used to identify potential study participants. From 2007 to 2010, 34,040 eligible subjects aged ≥50 years who resided in the Dong-gu district of Gwangju Metropolitan City in South Korea (35°N) were invited by telephone to participate in the study, and 9,260 subjects were subsequently enrolled (response rate: 27.2%; 3,713 males and 5,547 females). We selected 1,246 participants who were taking medication to treat type 2 diabetes. After excluding 71 participants because their data were incomplete; no vitamin D data (*n* = 58) or no HbA_1c_ data (*n* = 13), we included 1,175 subjects (563 males and 612 females) in the final analyses.

### Study measurements

Trained examiners interviewed all participants using a standardized questionnaire to assess smoking status, the use of anti-hypertensive medications, and the duration of diabetes. Height was measured to the nearest 0.1 cm and weight to the nearest 0.1 kg. The body mass index (BMI) was calculated by dividing a subject’s weight (in kg) by the height squared (in m^2^). Blood samples were drawn from antecubital veins following 12-h overnight fasts; the sera were separated within 30 min, and samples were stored at –70°C prior to analysis. The levels of fasting blood glucose, total cholesterol, triglycerides, and high-density lipoprotein (HDL) cholesterol were measured using enzymatic methods on an automatic analyzer (Hitachi-7600; Hitachi, Ltd., Tokyo, Japan). Low-density lipoprotein (LDL) cholesterol was measured as described by Friedewald *et al*. [15], except when the triglyceride level exceeded 400 mg/dL. HbA_1c_ levels were analyzed by high-performance liquid chromatography using the VARIANT II system (Bio-Rad, Hercules, CA, USA).

#### Measurement of 25(OH)D and PTH levels

Serum 25(OH)D and PTH levels were measured with the aid of a microparticle immunoassay system detecting chemiluminescence (Architext i2000; Abbott Diagnostics, Abbott Park, IL, USA). In a comparative study of automated immunoassays and liquid chromatography-tandem mass spectrometry methods (LC-MS/MS) [16], the Abbott 25(OH)D assay was comparable to LC-MS/MS; the concordance correlation coefficient was 0.85, and the mean bias was 4.56 ng/mL. The coefficient of variation for the total analytic precision of this assay was < 10% for 25(OH)D. According to the manufacturer's specifications, the assay has 105% cross-reactivity with 25(OH)D3, 82% cross-reactivity with 25(OH)D2, 12.6% cross-reactivity with 1,25(OH)_2_D3, 112% cross-reactivity with 24,25(OH)_2_D3, and minimal cross-reactivity with 3-epimer of 25(OH)D3 (2.7%).

### Statistical analysis

Data are presented as means ± standard deviations (SDs) or as percentages by the 25(OH)D and PTH quartiles because no consensus about cut-off value for 25(OH)D and PTH has been achieved [17,18,19]. The 25(OH)D and PTH data were not normally distributed; thus, to approximate normal distributions, the 25(OH)D and PTH data were log-transformed. In addition, the 25(OH)D and PTH quartiles are based on the distribution of the log-25(OH)D and log-PTH values (25[OH]D quartiles: Quartile 1 [Q1]; <12.5 ng/mL, Quartile 2 [Q2]; 12.5–15.2 ng/mL, Quartile 3 [Q3]; 15.3–19.0 ng/mL, and Quartile 4 [Q4]; >19.0 ng/mL, PTH quartiles: Q1; <26.2 pg/mL, Q2; 26.2–34.8 pg/mL, Q3; 34.9–46.1 pg/mL, and Q4; >46.1 pg/mL). Analysis of covariance (ANCOVA) (three models) was used to compare the mean HbA_1c_ and fasting glucose levels by the 25(OH)D and PTH quartiles. Model 1 was adjusted for the month of blood collection, sex, and age. Model 2 included the variables of Model 1 and smoking status, the use of anti-hypertensive medication, the duration of diabetes, and total cholesterol, triglyceride, and HDL cholesterol levels. Model 3 included the variables of Model 2 and the log-PTH and log-25(OH)D data. All statistical analyses were performed using SPSS software version 15.0 (SPSS, Inc., Chicago, IL, USA). A p-value <0.05 was considered to reflect statistical significance.

## Results

### Characteristics of subjects according to 25(OH)D quartiles

The characteristics of the 1,175 subjects (563 males and 612 females) included in the present study are listed in Table 1. A higher 25(OH)D level tended to be associated with male gender, a greater height, a heavier weight, smoking status, a greater level of physical activity, and lower levels of total cholesterol, triglycerides, LDL cholesterol, HDL cholesterol, HbA_1c_, and PTH. 25(OH)D levels showed a seasonal pattern being higher in June and July than in April and May.

**Table 1:** General characteristics of the subjects according to 25(OH)D quartiles.

Variable	25(OH)D (ng/mL)					*p* value
	Q1(≤12.40)	Q2(12.50 to15.10)	Q3(15.20 to19.00)	Q4(≥19.10)	Total	
*N* (%)	295(25.1)	288(24.5)	296(25.2)	296(25.2)	1175(100.0)	*-*
Male (%)	72(24.4)	102(35.4)	166(56.1)	223(75.3)	563(47.9)	<0.001
Age (years)	68.0±8.0	67.8±6.9	67.2±7.4	67.1±7.5	67.5±7.5	0.350
Diabetic duration (years)	9.8±8.5	8.9±7.8	9.7±8.7	10.2±9.2	9.6±8.6	0.328
Month of blood collection						<0.001
April	91(30.8)	69(24.0)	54(18.2)	36(12.2)	250(21.3)	
May	117(39.7)	102(35.4)	93(31.4)	80(27.0)	392(33.4)	
June	68(23.1)	79(27.4)	97(32.8)	114(38.5)	358(30.5)	
July	19(6.4)	38(13.2)	52(17.6)	66(22.3)	175(14.9)	
Height (cm)	155.9±7.7	157.0±8.4	160.3±8.6	163.1±8.0	159.1±8.7	<0.001
Weight (kg)	60.3±9.0	62.0±9.9	64.1±10.1	65.6±8.6	63.0±9.6	<0.001
BMI (kg/m^2^)	24.8±3.2	25.1±3.1	24.9±3.1	24.6±2.4	24.9±3.0	0.241
Current smoker (%)	22(7.5)	32(11.1)	48(16.2)	48(16.3)	150(12.8)	<0.001
^a^Physically active (%)	37(13.5)	42(15.0)	65(22.3)	82(28.2)	226(19.9)	<0.001
Hypertensive medication (%)	174(59.0)	167(58.0)	178(60.1)	163(55.1)	682(58.0)	0.633
Total cholesterol (mg/dL)	204.6±49.3	194.8±40.0	181.5±41.2	175.9±37.5	189.1±43.6	<0.001
Triglycerides (mg/dL)	185.6±219.1	166.4±121.3	146.1±88.4	147.7±92.6	161.4±141.4	0.002
LDL cholesterol (mg/dL)	120.6±40.9	115.1±35.6	104.8±37.2	100.4±33.9	110.1±37.8	<0.001
HDL cholesterol (mg/dL)	50.6±11.3	48.9±12.6	48.7±11.5	46.4±10.2	48.6±11.5	<0.001
Fasting glucose (mg/dL)	145.7±43.5	144.4±44.4	142.7±39.0	142.2±37.8	143.7±41.2	0.725
Hemoglobin A_1c_ (%)	7.5±1.5	7.2±1.3	7.1±1.3	7.2±1.3	7.3±1.4	0.004
PTH (pg/mL)	46.2±36.9	41.2±19.3	37.2±21.3	33.5±13.4	39.5±24.7	<0.001

All values are given as *n* (%) or mean ± standard deviation.

BMI, body mass index; 25(OH)D, 25-hydroxyvitamin D; LDL, low-density lipoprotein; HDL, high-density lipoprotein; PTH, parathyroid hormone

^a^Subjects who performed 30 min or more of moderate activity at least 5 days a week or 20 min of vigorous physical activity at least 3 days a week were regarded as doing physical activity

### Characteristics of subjects according to PTH quartiles

Subject characteristics by PTH quartile are listed in Table 2. A higher PTH level tended to be associated with female gender, older age, a lower height, a higher BMI, non-smoking status, greater use of anti-hypertensive medications, lower levels of fasting glucose, HbA_1c_, and 25(OH)D.

**Table 2:** General characteristics of the subjects according to PTH quartiles.

Variable	PTH (pg/mL)					*p* value
	Q1(≤26.1)	Q2(26.2 to 34.8)	Q3(34.9 to 46.1)	Q4(≥46.2)	Total	
*N* (%)	294(25.0)	293(25.0)	295(25.1)	292(24.9)	1174(100.0)	
Male (%)	163(55.4)	139(47.4)	140(47.5)	121(41.4)	563(48.0)	0.009
Age (years)	67.1±7.3	66.2±7.6	67.7±7.2	69.1±7.5	67.5±7.5	<0.001
Diabetic duration (years)	10.2±9.0	9.8±8.1	9.0±8.0	9.5±9.1	9.6±8.6	0.328
Month of blood collection						0.494
April	60(20.4)	64(21.8)	58(19.7)	67(22.9)	249(21.2)	
May	91(31.0)	88(30.0)	105(35.6)	108(37.0)	392(33.4)	
June	96(32.7)	90(30.7)	89(30.2)	83(28.4)	358(30.5)	
July	47(16.0)	51(17.4)	43(14.6)	34(11.6)	175(14.9)	
Height (cm)	160.2±8.6	159.3±8.8	158.9±8.7	158.0±8.5	159.1±8.7	0.018
Weight (kg)	62.7±9.3	63.2±9.2	63.5±10.3	62.6±9.7	63.0±9.6	0.600
BMI (kg/m^2^)	24.4±2.7	24.9±2.9	25.1±3.0	25.1±3.2	24.9±3.0	0.010
Current smoker (%)	56(19.0)	32(11.0)	32(10.8)	30(10.3)	150(12.8)	0.003
^a^Physically active (%)	69(24.3)	60(21.4)	46(16.1)	51(17.8)	226(19.9)	0.068
Hypertensive medication (%)	141(48.0)	158(53.9)	188(63.7)	194(66.4)	681(58.0)	<0.001
Total cholesterol (mg/dL)	190.3±46.7	188.7±43.6	189.0±42.3	188.4±42.1	189.1±43.7	0.955
Triglycerides (mg/dL)	164.6±154.8	166.0±178.4	157.9±114.5	157.4±106.4	161.5±141.5	0.831
LDL cholesterol (mg/dL)	110.8±38.3	110.1±36.5	110.2±37.9	109.3±38.7	110.1±37.8	0.974
HDL cholesterol (mg/dL)	48.4±11.2	47.9±10.7	48.7±11.5	49.4±12.7	48.6±11.5	0.456
Fasting glucose (mg/dL)	146.8±44.3	144.7±40.7	145.4±39.2	137.9±39.8	143.7±41.2	0.042
Hemoglobin A_1c_ (%)	7.4±1.3	7.3±1.5	7.2±1.3	7.1±1.3	7.3±1.4	0.033
25(OH)D (ng/mL)	17.7±5.8	16.3±5.5	16.3±5.3	14.4±4.5	16.2±5.4	<0.001

All values are given as *n* (%) or mean ± standard deviation.

BMI, body mass index; PTH, parathyroid hormone; LDL, low-density lipoprotein; HDL, high-density lipoprotein; 25(OH)D, 25-hydroxyvitamin D

^a^Subjects who performed 30 min or more of moderate activity at least 5 days a week or 20 min of vigorous physical activity at least 3 days a week were regarded as doing physical activity

### Comparison of mean HbA_1c_ and fasting glucose values according to 25(OH)D quartiles

The mean HbA_1c_ values (95% confidence intervals [CIs]) by 25(OH)D quartiles are listed in Table 3. After adjusting for the month of blood collection, sex, age, BMI, smoking status, the extent of physical activity, the use of anti-hypertensive medications, the duration of diabetes, and the levels of total cholesterol, triglycerides, HDL cholesterol, and the log-PTH level (Model 3), the association between HbA_1c_ and 25(OH)D levels remained significant (Q1: 7.47% [7.30–7.63], Q2: 7.25% [7.09–7.40], Q3: 7.17% [7.02–7.32], Q4: 7.19% [7.02–7.35]; *p* for trend = 0.021, *p* for between groups = 0.050). The mean fasting glucose levels did not vary by the 25(OH)D quartile after adjusting for all covariates and the log-PTH level (*p* for trend = 0.608, *p* for between groups = 0.945).

**Table 3:** Comparison of mean HbA_1c_ and fasting glucose according to 25(OH)D quartiles.

	25(OH)D level	Model 1	Model 2	Model 3
		Mean (95% CI)	Mean (95% CI)	Mean (95% CI)
Fasting glucose (mg/dL)	Quartile 1	145.1(140.2–150.0)	144.4 (139.5–149.3)	144.7 (139.7–149.7)
	Quartile 2	144.4(139.7–149.2)	144.8 (140.1–149.5)	145.1 (140.4–149.8)
	Quartile 3	142.7(138.1–147.4)	144.1 (139.5–148.7)	143.9 (139.3–148.4)
	Quartile 4	142.7(137.8–147.7)	143.6 (138.7–148.4)	143.0 (138.1–147.9)
	*p* for trend	0.468	0.787	0.608
	*p* for between groups	0.893	0.988	0.945
Hemoglobin A_1c_ (%)	Quartile 1	7.48 (7.32–7.64)*	7.45 (7.29–7.61)	7.47 (7.30–7.63)*
	Quartile 2	7.20 (7.04–7.36)	7.24 (7.08–7.39)	7.25 (7.09–7.40)
	Quartile 3	7.13 (6.98–7.29)	7.18 (7.03–7.33)	7.17 (7.02–7.32)
	Quartile 4	7.19 (7.03–7.36)	7.20 (7.05–7.36)	7.19 (7.02–7.35)
	*p* for trend	0.018	0.043	0.021
	*p* for between groups	0.018	0.083	0.050

Model 1, Adjusted by month of blood collection, sex and age

Model 2, Adjusted by Model 1 plus BMI, smoking, physical activity, hypertension medications, diabetic duration, total cholesterol, triglyceride and HDL cholesterol

Model 3, Adjusted by Model 2 plus log-PTH

*p<0.005 compared with Quartile 3 with the Bonferroni adjustment for multiple comparison

### Comparison of mean HbA_1c_ and fasting glucose values according to PTH quartiles

The mean HbA_1c_ values (95% CIs) by PTH quartiles are listed in Table 4. After adjusting for the covariates described above and the log-25(OH)D value (Model 3), the association between HbA_1c_ and PTH levels became significant (Q1: 7.35% [7.19–7.51], Q2: 7.34% [7.19–7.50], Q3: 7.28% [7.13–7.43], Q4: 7.09% [6.94–7.24]; *p* for trend = 0.022, *p* for between groups = 0.048). The mean fasting glucose value did not differ by PTH quartile after adjusting for all covariates and the log-25(OH)D value (*p* for trend = 0.104, *p* for between groups = 0.088).

**Table 4:** Comparison of mean HbA_1c_ and fasting glucose according to PTH quartiles.

	PTH level	Model 1	Model 2	Model 3
		Mean (95% CI)	Mean (95% CI)	Mean (95% CI)
Fasting glucose (mg/dL)	Quartile 1	146.7(142.0–151.4)	146.2 (141.6–150.9)	146.2 (141.6–150.9)
	Quartile 2	144.0(139.3–148.6)	144.0 (139.4–148.7)	144.0 (139.4–148.7)
	Quartile 3	145.5(140.9–150.1)	147.1 (142.5–151.7)	147.1 (142.5–151.7)
	Quartile 4	138.5(133.8–143.2)	139.2 (134.6–143.9)	139.2 (134.6–143.9)
	*p* for trend	0.033	0.092	0.104
	*p for between groups*	0.080	0.080	0.088
Hemoglobin A_1c_ (%)	Quartile 1	7.39(7.24–7.55)*	7.32 (7.16–7.47)	7.35 (7.19–7.51)*
	Quartile 2	7.32(7.17–7.48)	7.35 (7.19–7.50)	7.34 (7.19–7.50)
	Quartile 3	7.21(7.05–7.36)	7.28 (7.13–7.43)	7.28 (7.13–7.43)
	Quartile 4	7.08(6.92–7.23)	7.12 (6.97–7.27)	7.09 (6.94–7.24)
	*p* for trend	0.003	0.067	0.022
	*p for between groups*	0.021	0.124	0.048

Model 1, Adjusted by month of blood collection, sex and age

Model 2, Adjusted by Model 1 plus BMI, smoking, physical activity, hypertension medications, diabetic duration, total cholesterol, triglyceride and HDL cholesterol

Model 3, Adjusted by Model 2 plus log-25(OH)D

*p<0.005 compared with Quartile 4 with the Bonferroni adjustment for multiple comparison

### Mean HbA_1c_ values according to the 25(OH)D and PTH quartiles

**Fig 1:**
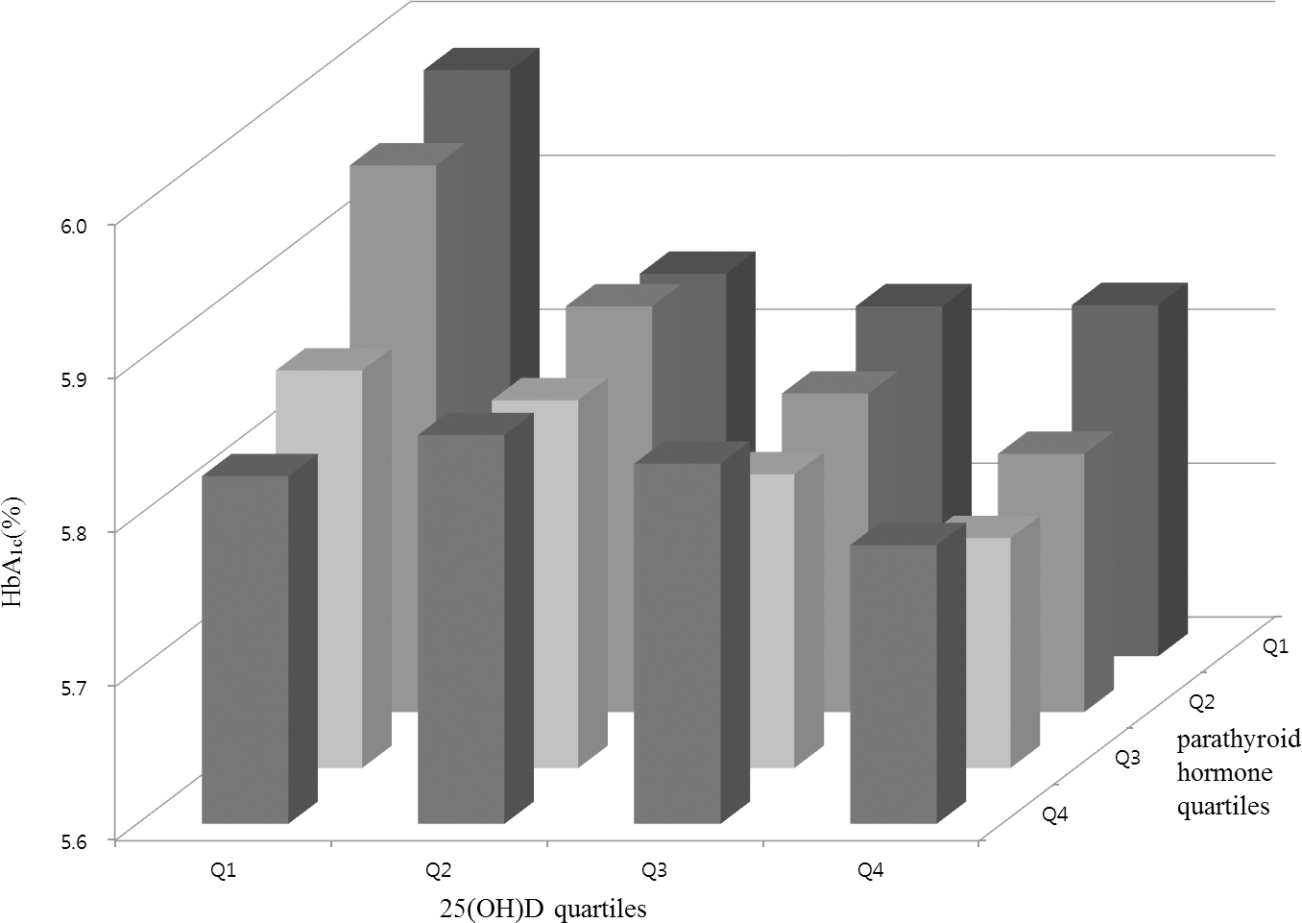
Mean HbA_1c_ levels by the 25(OH)D and PTH quartiles.

Fig 1 shows how the mean HbA_1c_ value varied among the 25(OH)D and PTH quartiles. The mean HbA_1c_ value tended to decrease in the higher 25(OH)D and PTH quartiles and to increase in the lower quartiles.

## Discussion

Inverse associations were evident between the HbA_1c_, 25(OH)D and PTH levels in Korean patients with type 2 diabetes. These associations remained significant after adjustment for other covariates, including the log-25(OH)D and log-PTH values. However, no association was evident between the fasting glucose level and either the 25(OH)D or PTH level.

Consistent with earlier reports, we found that the 25(OH)D level was influenced by month of blood collection [20], physical activity [21], lipid profiles [22,23] and PTH [24,25]. Unlike the findings of previous studies [20,26], BMI was not associated with the 25(OH)D level. The Longitudinal Aging Study conducted in Amsterdam [27] sought associations between 25(OH)D levels and bodily composition variables, including BMI, waist-to-hip ratio, waist circumference, total body fat, and total body fat percentage. The association of the 25(OH)D level with the total body fat percentage was stronger than were the associations with other bodily composition variables. This indicates that adiposity, and not simply body weight or BMI per se, is associated with the 25(OH)D concentration [27]. When we analyzed association between the 25(OH)D levels with body fat percentage in our data (data are not shown), body fat percentage is inversely associated with 25(OH)D quartiles (Q1: 34.6±7.2%, Q2: 33.6±7.3%, Q3: 31.0±7.7%, Q4: 29.2±6.3%, p<0.001) like previous study [27].

Many studies have explored associations between 25(OH)D levels and the prevalence of diabetes; lower 25(OH)D levels were associated with a higher prevalence of type 2 diabetes [10,28,29]. However, any relationship between the 25(OH)D level and the HbA_1c_ level remains unclear. Some authors reported positive relationships between the 25(OH)D level and the HbA_1c_ level [30]; others presented no association [12]; the others reported inverse associations [10,11,31].

We found that the association between the HbA_1c_ and 25(OH)D levels was significant, but no association was apparent between the fasting glucose and 25(OH)D levels. In a previous study with 668 older subjects [10], an association was evident between 25(OH)D and HbA_1c_ levels, but not between 25(OH)D and fasting glucose levels (as also found by us). The 25(OH)D level is related to the HbA_1c_ level but not to the fasting glucose level, because the HbA_1c_ level is a long-term marker of glycemic control, reflecting changes that take place over about 2–3 months [32]. Such changes are difficult to capture using single measurements of fasting glucose levels.

We found that, after adjustment for the log-PTH level, the association between the HbA_1c_ and 25(OH)D levels remained significant. To the best of our knowledge, only a few previous studies have explored the relationship between the 25(OH)D level and the extent of glycemic control after adjusting for the PTH level. In the NHANES study (2001–2006) on 3,958 subjects [33], associations of the 25(OH)D level with fasting glucose and insulin levels were independent of the serum PTH concentration. In another study using NHANES data collected from 2003–2006 [11], an analysis of 9,773 subjects showed that the 25(OH)D level was inversely associated with the HbA_1c_ level after adjusting for other covariates including the PTH level, which is similar to what we found. 1,25 dihydroxyvitamin D3 (1,25[OH]_2_D3) regulates insulin synthesis/secretion by controlling calcium homeostasis via a mechanism involving PTH [34,35], and it may also (in a PTH-independent manner) influence glucose metabolism via activation of VDRs on pancreatic β-cells [36] and protects β-cells from immune attack [37]. Recently, it has been shown that vitamin D3 can be transformed by the steroidogenic enzyme cytochrome P450scc (CYP11A) into not only 25(OH)D3 and 1,25(OH)_2_D3, but also the novel metabolites 20(OH)D3, 22(OH)D3, 20,22(OH)_2_D3, and 20,23(OH)_2_D3 [38,39]. The major product of CYP11A acting on vitamin D3, 20(OH)D3 is noncalcemic [40], and it may regulate 1,25(OH)_2_D3 production in a PTH-independent manner [41].

We found that the HbA_1c_ level was inversely associated with the PTH level, independently of 25(OH)D status. This is surprising; previous studies associated elevated PTH levels with glucose intolerance and insulin insensitivity [42,43]. This discrepancy may not be attributable to differences in PTH distribution. The mean (with standard deviation [SD]) level of PTH in one study was 42.1±15.7 pg/mL [43], and the mean (with standard error [SE]) level in another study was 40.11(1.74) pg/mL [42], whereas the mean ± SD and mean (SE) levels in our study were 39.5±24.7 pg/mL and 39.5 (0.72) pg/mL, respectively. The precise mechanisms how increased PTH is associated with decreased HbA_1c_ level are not clear. Under normal physiological conditions, low levels of vitamin D should stimulate PTH secretion, and low levels of PTH should increase calcium uptake from both the gut and skeleton [44]. Therefore, high-level PTH reflects a low calcium level, which may improve glucose-tolerance [45], and ameliorate both insulin sensitivity and insulin secretion [46].

The primary strengths of this study lie in its population based design and use of a relatively large sample size, which minimized selection bias and provided sufficient statistical power. However, a number of limitations should also be considered. First, the present study employed a cross-sectional design. Second, it was comparatively limited in its ability to explain seasonal changes in 25(OH)D, partly due to a lack of information regarding sun exposure during seasonal cycles. Finally, only a single serum 25(OH) D measurement was taken, and the data therefore reflect only a single point in time rather than long-term exposure.

## Conclusion

Inverse associations were evident between HbA_1c,_ 25(OH)D and PTH levels in Korean patients with type 2 diabetes. Such associations remained significant after adjustment for covariates, including the log-25(OH)D and log-PTH values.
